# 

**Published:** 1883-06

**Authors:** 


ESTABLISHED IN 1855.

OLD SERIES. Vol. XXI—No. 3. JUNE, 1883. NEW SERIES Vol. II—No. 9

### ATLANTA

### MEDICAL REGISTER.

*(ATLANTA MEDICAL AND SURGICAL JOURNAL.)*

S.G.O. INDEXED

EDITED BY

J. H. LOGAN, A.M., M.D.

H. H. DICKSON, PUBLISHER. TERMS, $2.50 A YEAR IN ADVANCE

ATLANTA, GEORGIA:

*H. H. DICKSON, BOOK AND JOB PRINTER AND BOOKBINDER.*

1883.

